# Amurensin G Sensitized Cholangiocarcinoma to the Anti-Cancer Effect of Gemcitabine via the Downregulation of Cancer Stem-like Properties

**DOI:** 10.3390/nu16010073

**Published:** 2023-12-25

**Authors:** Yun-Jung Na, Hong Kyu Lee, Kyung-Chul Choi

**Affiliations:** Laboratory of Biochemistry and Immunology, College of Veterinary Medicine, Chungbuk National University, Cheongju 28644, Chungbuk, Republic of Korea; sky1105a@naver.com (Y.-J.N.); hklee83@cbnu.ac.kr (H.K.L.)

**Keywords:** cholangiocarcinoma, amurensin G, gemcitabine, cancer stem cell, chemoresistance

## Abstract

Cholangiocarcinoma (CCA) is a malignant biliary tract tumor with a high mortality rate and refractoriness to chemotherapy. Gemcitabine is an anti-cancer chemotherapeutic agent used for CCA, but the efficacy of gemcitabine in CCA treatment is limited, due to the acquisition of chemoresistance. The present study evaluated the chemosensitizing effects of Amurensin G (AMG), a natural sirtuin-1 inhibitor derived from *Vitis amurensis*, in the SNU-478 CCA cells. Treatment with AMG decreased the SNU-478 cell viability and the colony formation ability. Annexin V/ Propidium iodide staining showed that the AMG increased apoptotic death. In addition, AMG downregulated anti-apoptotic Bcl-2 expression, while upregulating pro-apoptotic cleaved caspase-3 expression. Treatment with AMG decreased the migratory ability of the cells in a wound healing assay and transwell migration assay. It was observed that AMG decreased the gemcitabine-induced increase in CD44^high^CD24^high^CD133^high^ cell populations, and the expression of the Sox-2 protein was decreased by AMG treatment. Co-treatment of AMG with gemcitabine significantly enhanced the production of reactive oxygen species, as observed through mitochondrial superoxide staining, which might be associated with the downregulation of the Sirt1/Nrf2 pathway by AMG. These results indicate that AMG enhances the chemotherapeutic ability of gemcitabine by downregulating cancer stem-like properties in CCA cells. Hence, a combination therapy of AMG with gemcitabine may be an attractive therapeutic strategy for cholangiocarcinoma.

## 1. Introduction

Cholangiocarcinoma (CCA) is a malignant biliary tract tumor with a poor prognosis. Despite significant advances in diagnosis and therapy, the 5-year survival rate of CCA is still less than 20%. Because CCA has no clinical symptoms and no specific diagnostic indicators in the early stage, CCA is commonly diagnosed in the advanced stages. Surgical resection remains the mainstay of therapy, but recurrences are common, due to incomplete tumor removal [1,2]. The adjuvant chemotherapy with gemcitabine and cisplatin after CCA resection may reduce recurrence risk and improve survival [3,4]. However, the efficacy of these adjuvant chemotherapies for CCA treatment is limited, due to the acquisition of resistance, and relapse occurs frequently.

Gemcitabine inhibits cell division and the growth of cancer cells by interfering with DNA synthesis [5]. However, persistent treatment of gemcitabine, in turn, exerts drug resistance and toxicity through its effects on the tumor microenvironment and epithelial-mesenchymal transition (EMT) in cancer cells [5,6]. Drug resistance induced by gemcitabine is caused by various factors, including the high expression of drug efflux pumps and the upregulation of cancer stem-like cells (CSCs) via the activation of the Hedgehog, Wnt, and Notch pathways [7]. CSCs are subpopulations of undifferentiated cancer cells within the tumor that are responsible for tumor initiation, progression, and metastasis [8]. In addition, CSCs mediate EMT, stemness, and chemoresistance, thereby causing relapse in tumors [9]. Therefore, various approaches have been tested to sensitize cancer cells to conventional chemotherapeutics, through CSC downregulation.

Amurensin G (AMG), a member of the amurensis family, is a resveratrol trimer and is known to derive from specific plants, including *Phellodendron amurense*, some *Papaver* species, and *Vitis amurensis* [10,11]. AMG has been reported as a potent natural sirtuin 1 (Sirt1) inhibitor, and has shown neuroprotective effects in animal models [10,12,13]. In addition, AMG has anti-oxidant activity, induces apoptosis, and has anti-angiogenesis effects in cancer cells [14,15,16]. Previous studies have shown that AMG inhibits drug-resistant cell growth and reduces CSCs [14,15]. However, the anti-cancer effect of AMG in CCA and its related mechanisms have not been elucidated, to date. 

In the present study, we investigated the effects of AMG on CCA sensitization to the gemcitabine by confirming cancer stem-like cell properties, including apoptosis, colony formation, migratory ability, and ROS formation in the SNU-478 CCA cell line. This study suggests that AMG increases the anti-cancer effect mediated by gemcitabine, and combination treatment of AMG with gemcitabine may be a promising therapeutic strategy for CCA.

## 2. Materials and Methods

### 2.1. Cell Culture and Reagents

CCA (SNU-478) cell line was obtained from the Korean Cell Line Bank (KCLB; Seoul, Republic of Korea). The SNU-478 cells were maintained in Roswell Park Memorial Institute (RPMI) 1640 (Welgene, Gyeongsan, Republic of Korea), supplemented with 10% fetal bovine serum (FBS; R&D systems, Minneapolis, MN, USA), 1% antibiotic-antimycotic solution (Welgene) and 1% HEPES buffer solution (Welgene) in a humidified incubator with 5% CO_2_ at 37 °C. When the cell density was 70–80%, trypsinization was performed using 0.25% trypsin (Welgene). AMG, isolated from the stem of *Vitis amurensis*, according to the procedure previously described [13], was kindly provided by Prof. Dr. Yeon Hee Seong (Laboratory of Pharmacology, College of Veterinary Medicine, Chungbuk National University, Cheongju, Republic of Korea). Gemcitabine was purchased from Sigma-Aldrich (St. Louis, MO, USA).

### 2.2. Cell Viability Assay

Cell viability was measured using a Quanti-MAX water-soluble tetrazolium salt (WST) assay kit (Biomax, Seoul, Republic of Korea), according to the manufacturer’s instructions, with slight modifications. SNU-478 cells (4 × 10^3^ cells/well) were plated in a 96-well cell culture plate (Sarstedt, Nümbrecht, Germany). The cells were pre-incubated for 24 h, and AMG and/or gemcitabine were treated. After a further 72 h of incubation, the absorbance was measured using a Neo2 Hybrid Multimode Reader (Agilent Technologies, Inc., Santa Clara, CA, USA).

### 2.3. Colony Formation Assay

The ability of colony formation was evaluated using a colony formation assay, as described previously, with slight modifications [17]. CCA (SNU-478) cells (5 × 10^2^ cells/well) were seeded in 6-well cell culture plates. After 24 h of pre-incubation, the cells were treated with AMG (5 μM) and/or gemcitabine (1 nM). After 8 days of incubation, the cells were fixed with 4% paraformaldehyde and stained with 0.5% crystal violet (Sigma-Aldrich). The colony areas were measured using Image J software v.1.49. 

### 2.4. Cell Apoptosis Assay

The types of cell death were evaluated using Annexin V/Propidium iodide (PI) staining, according to the manufacturer’s instructions. SNU-478 cells (2 × 10^5^ cells/well) were plated in 6-well cell culture plates. After 24 h of incubation, the cells were co-incubated with AMG (5 μM) and/or gemcitabine (500 nM) for 48 h. After collecting the cells, they were reacted with Annexin V (BioLegend, San Diego, CA, USA) and propidium iodide (PI) (Invitrogen, Waltham, MA, USA). The samples were acquired using fluorescence-activated cell sorting (FACS) Symphony A3 (BD Biosciences, San Diego, CA, USA), and results were analyzed with the FlowJo Software v. 10.8.1 (TreeStar, San Carlos, CA, USA). 

### 2.5. Wound Healing Assay

The cell migratory ability was evaluated using the wound healing assay, as described previously, with slight modifications [18]. SNU-478 cells (5 × 10^5^ cells/well) were seeded in 6-well plates (SPL, Gyeonggi, Republic of Korea). After 24 h, cells were treated with 3.5 μg/mL Mitomycin C (Sigma-Aldrich) for 1.5 h, and scratched using a sterile plastic tip. After washing with Dulbecco’s Phosphate-Buffered Saline (DPBS, Welgene), the cells were treated with AMG (5 μM) and/or gemcitabine (100 nM) for 48 h. Images were acquired using the Ⅸ-73 inverted microscope (Olympus, Tokyo, Japan), and the scratch area was measured by CellSens Dimension 1.13 version (Olympus).

### 2.6. Transwell Migration Assay

The cell migratory ability was evaluated using the transwell migration assay, as described previously, with slight modifications [19]. SNU-478 cells (1 × 10^5^ cells/well) were seeded in the upper chamber (24-well, 8.0 μm pore membrane, Corning, Somerville, MA, USA). The upper chamber was treated with serum-free media with AMG (5 μM) and/or gemcitabine (100 nM), and the lower chamber was treated with media (10% FBS) with AMG (5 μM) and/or gemcitabine (100 nM). After 24 h, the surface of the upper chamber was fixed with 4% paraformaldehyde (Geneall, Seoul, Republic of Korea) and stained with 0.5% crystal violet (Sigma-Aldrich). The images of migrated cells were photographed using an (Olympus) Ⅸ-73 inverted microscope, and the number of migrated cells was counted using CellSens Dimension 1.13 version (Olympus). 

### 2.7. Flow Cytometry Analysis

The CD44, CD24, and CD133 populations were detected by FACS analysis. SNU-478 cells (2 × 10^5^ cells/well) were plated in 6-well cell culture plates (Sarstedt). After 24 h, the cells were treated with AMG (5 μM) and/or gemcitabine (500 nM) for 48 h. After harvesting, cells were stained with allophycocyanin (APC)-labeled anti-human CD133 (BioLegend), phycoerythrin (PE)-labeled anti-human CD24 (BioLegend), and fluorescein isothiocyanate (FITC)-labeled anti-human CD44 (BioLegend) (Table 1). Stained cells were acquired using FACS Symphony A3 (BD Life Sciences Bioscience, San Diego, CA, USA) and results were analyzed using the FlowJo Software v. 10.8.1 (TreeStar, San Carlos, CA, USA). 

### 2.8. Mitochondrial Superoxide (MitoSOX) Assay

MitoSOX^TM^ staining was performed according to the manufacturer’s instructions, with slight modifications. SNU-478 cells (6 × 10^3^ cells/well) were seeded in a 96-well cell culture plate. After 24 h, cells were treated with AMG (5 μM) and/or gemcitabine (500 nM) for 48 h. Then, staining was performed with MitoSOX^TM^ (5 μM) (Invitrogen) and Hoechst 33342 (10 μg/mL) (Sigma-Aldrich). Images of stained cells were captured by Lionheart^TM^ FX Automated Microscope (BioTek Instruments Inc., Winooski, VT, USA), and analyzed using Gen5 v 3.14.03 (Agilent Technologies, Inc.).

### 2.9. Western Blot Assay

The proteins from SNU-478 cells were extracted from cells using PRO-PREP^TM^ (iNtRON Biotechnology, Inc., Seongnam, Republic of Korea). Protein concentration was determined by bicinchoninic acid assay (Sigma-Aldrich), according to the manufacturer’s instructions. Western blot was performed using the JESS^TM^ Simple Western automated nano-immunoassay system (ProteinSimple, San Jose, CA, USA). The antibodies used were the following: Sirt1, Kelch-like ECH-associated protein 1 (Keap1), nuclear factor erythroid-2 related factor 2 (Nrf2), Sex determining region Y-box 2 (Sox-2), cleaved caspase-3 (c-cas3) (Cell Signaling Technology, Danvers, MA, USA), B-cell lymphoma-2 (Bcl-2; Biolegend), β-actin and glyceraldehyde-3-phosphate dehydrogenase (GAPDH) (Abcam, Cambridge, UK) (Table 1). The band images were captured by Compass Simple Western software (v 6.0.0, ProteinSimple), and the intensities of the target protein were measured using Image J software v.1.49.

### 2.10. Statistical Analysis

All data were presented as means ± standard deviation (S.D.). Statistical significances of all the data were analyzed using one-way analysis of variance (ANOVA), followed by a post hoc Dunnett’s test, using the GraphPad Prism 5.01 software (GraphPad Software Inc., San Diego, CA, USA). A *p*-value < 0.05 is considered statistically significant.

## 3. Results

### 3.1. AMG Decreases Cell Growth in SNU-478 Cells

**Figure 1:**
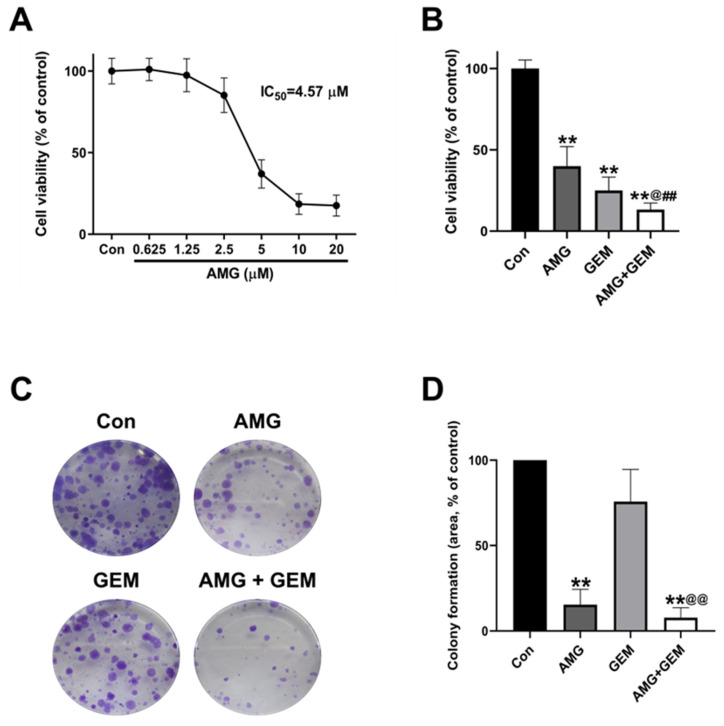
Effects of co-treatment of AMG and gemcitabine on cell viability and proliferation of SNU-478 cells. (**A**) The representative graph shows the changes in cell viability by AMG, for 72 h. (**B**) The graph shows the change in cell viability by AMG and/or gemcitabine, for 72 h. (**C**) The representative images show colony formation in SNU-478 cells. (**D**) Quantification of colony-forming area. Data are presented as means ± S.D. from at least three independent experiments. ** *p* < 0.01 vs. Con; ^@^ *p* < 0.05, ^@@^ *p* < 0.01 vs. GEM; ^##^ *p* < 0.01 vs. AMG (Dunnet’s test). Con, control; AMG, amurensin G; GEM, gemcitabine.

To evaluate the effects of AMG on cell viability, a WST assay was performed on SNU-478 cells. Based on preliminary experimental results, gemcitabine was chosen at an appropriate concentration of 500 nM. AMG decreased cell viability, with half-maximal inhibitory concentration (IC_50_) values of 4.57 μM in the SNU-478 cells (Figure 1A). AMG or gemcitabine significantly decreased cell viability. Combination treatment of AMG with gemcitabine presented additively reduced cell viability (Figure 1B). A colony formation assay was performed to confirm the effect of AMG and gemcitabine on cell proliferation. The use of gemcitabine 500 nM caused almost all cell death, preventing the continuation of the experiment. Therefore, we used an appropriate concentration of gemcitabine 1 nM. AMG (5 μM) significantly reduced the colony-forming area compared with the control, while gemcitabine (1 nM) treatment showed no significant difference in the colony-forming area. Combination treatment of AMG (5 μM) with gemcitabine (1 nM) significantly decreased the colony area compared with the gemcitabine-alone treatment (Figure 1C,D). These results indicated that the AMG reduced cell survival and proliferation, and co-treatment of AMG with gemcitabine additively inhibits cell survival and proliferation.

### 3.2. Combination Treatment of AMG with Gemcitabine Induces Apoptosis in SNU-478 Cells

**Figure 2:**
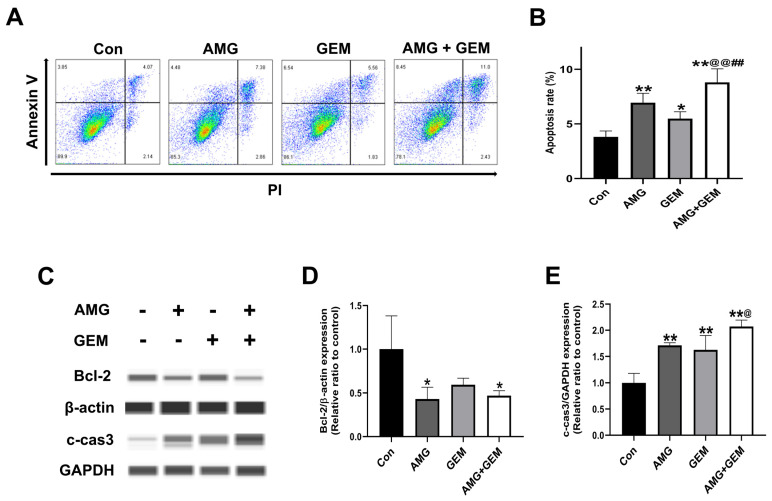
Effects of co-treatment of AMG and gemcitabine on types of cell death in SNU-478 cells. (**A**) Representative flow cytometry plots with Annexin V/PI staining for apoptosis. (**B**) Percentage of apoptotic cells. (**C**) The protein expression of Bcl-2 and c-cas3 was detected by Western blotting after the treatment for 48 h. (**D**) The protein expression of Bcl-2 was normalized with β-actin. (**E**) The protein expression of c-cas3 was normalized with GAPDH. Data are presented as means ± S.D. from at least three independent experiments. * *p* < 0.05, ** *p* < 0.01 vs. Con; ^@^ *p* < 0.05, ^@@^ *p* < 0.01 vs. GEM; ^##^ *p* < 0.01 vs. AMG (Dunnet’s test). Con, control; AMG, amurensin G; GEM, gemcitabine.

Annexin V/PI staining was used to investigate whether AMG induces apoptosis. AMG (5 μM) and gemcitabine (500 nM) significantly increased the Annexin V^+^ population, compared with the control. Combination treatment of AMG (5 μM) with gemcitabine (500 nM) significantly increased the Annexin V^+^ population, compared with all other groups. (Figure 2A,B). In addition, the expression of apoptosis-associated protein was examined using Western blot (Figure 2C). AMG treatment significantly reduced the level of Bcl-2, compared with the control. The expression level of Bcl-2 in the co-treatment of AMG with the gemcitabine group was lower than that of the control group. The level of c-cas3 in the combination treatment of AMG with the gemcitabine group was significantly higher than that of the control group (Figure 2D,E). These results indicated that AMG and gemcitabine co-treatment increased apoptotic cell death.

### 3.3. AMG Suppresses the Migratory Ability in SNU-478 Cells

**Figure 3:**
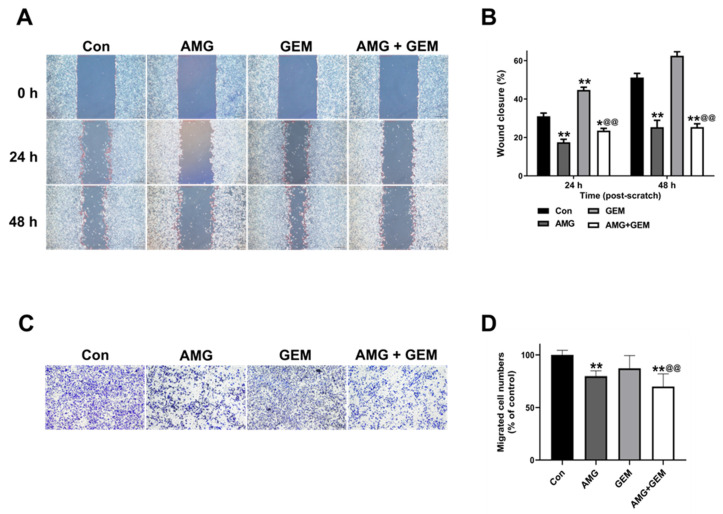
Effect of co-treatment of AMG and gemcitabine on cell migratory ability in SNU-478 cells. (**A**) The representative images of a wound healing assay in SNU-478 cells. Bar = 500 μm. (**B**) Quantification of wound-healing scratch area. (**C**) The representative images of a transwell migration assay in SNU-478 cells. Bar = 200 μm. (**D**) Quantification of transwell migrated cells. Data are presented as means ± S.D. from at least three independent experiments. * *p* < 0.05, ** *p* < 0.01 vs. Con; ^@@^ *p* < 0.01 vs. GEM (Dunnet’s test). Con, control; AMG, amurensin G; GEM, gemcitabine.

To evaluate the effect of AMG on the migratory ability of SNU-478 cells, a wound healing assay and transwell migration assay were performed. The use of gemcitabine 500 nM caused almost all cell death, preventing the continuation of the experiment. Therefore, we used an appropriate concentration of gemcitabine 100 nM. At 24 h, AMG (5 μM) significantly decreased wound closure, compared with the control, while gemcitabine (100 nM) significantly increased wound closure, compared with the control. Combination treatment of AMG (5 μM) with gemcitabine (100 nM) significantly decreased wound closure, compared with gemcitabine-alone treatment. At 48 h, AMG (5 μM) decreased wound closure compared with control, and wound closure of gemcitabine (100 nM) showed no significant difference. Combination treatment of gemcitabine (100 nM) with AMG (5 μM) significantly reduced wound closure, compared with gemcitabine-alone treatment (Figure 3A,B). In the transwell migration assay, AMG (5 μM) decreased the number of migrated cells when compared with the control, while gemcitabine (100 nM) showed no significant difference. Combination treatment of AMG (5 μM) with gemcitabine (100 nM) significantly decreased the number of migrated cells, compared with gemcitabine-alone treatment (Figure 3C,D). These results indicated that the migratory ability is inhibited by the treatment of AMG.

### 3.4. AMG Inhibits Cancer Stem-like Cell Properties in SNU-478 Cells

**Figure 4:**
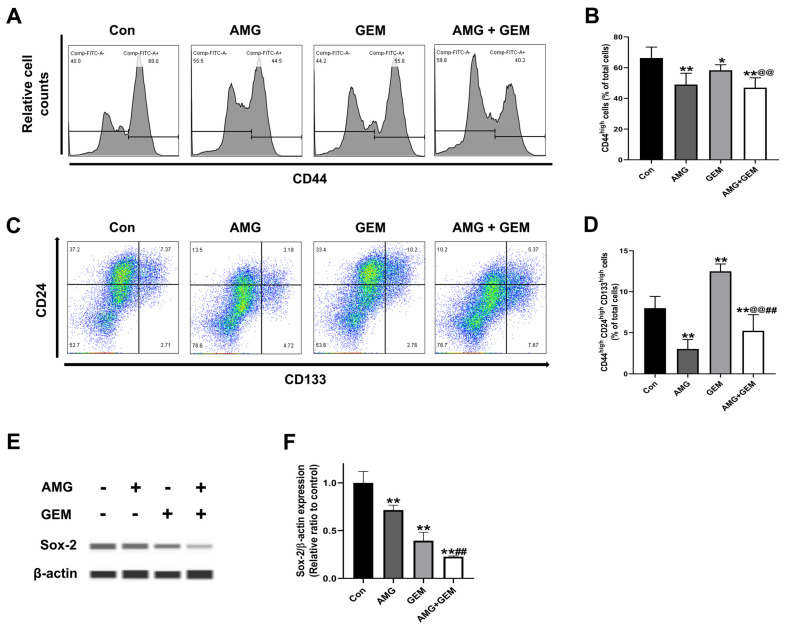
Effect of co-treatment of AMG and gemcitabine on cancer stem-like cell properties in SNU-478 cells. (**A**) Representative histogram of CD44. (**B**) The graph shows the percentage of CD44^high^ populations. (**C**) Representative plot of CD24 and CD133 among CD44^high^ cells. (**D**) The graph shows the percentage of CD44^high^CD24^high^CD133^high^ populations. (**E**) The protein expression of Sox-2 was detected using Western blotting. (**F**) The Sox-2 protein expression was normalized with β-actin. Data are presented as means ± S.D. from at least three independent experiments. * *p* < 0.05, ** *p* < 0.01 vs. Con; ^@@^ *p* < 0.01 vs. GEM; ^##^ *p* < 0.01 vs. AMG (Dunnet’s test). Con, control; AMG, amurensin G; GEM, gemcitabine.

To confirm the effect of AMG treatment in inhibiting CSCs, cancer stem-like cell subpopulations were assessed using FACS. AMG (5 μM) or gemcitabine (500 nM) decreased the CD44^high^ population, compared with the control. Combination treatment of AMG (5 μM) with gemcitabine (500 nM) significantly decreased the CD44^high^ population, compared with gemcitabine-alone treatment (Figure 4A,B). AMG (5 μM) significantly decreased the CD44^high^CD24^high^CD133^high^ populations compared with the control, while gemcitabine (500 nM) single treatment significantly increased CD44^high^CD24^high^CD133^high^ populations, compared with the control. Combination treatment of AMG (5 μM) with gemcitabine (500 nM) significantly decreased CD44^high^CD24^high^CD133^high^ populations when compared with other groups (Figure 4C,D). In addition, the expression of Sox-2 was examined using Western blot (Figure 4E). The expression level of Sox-2 in the AMG and gemcitabine co-treatment group was lower than that of the control group (Figure 4F). These results indicated that co-treatment of AMG with gemcitabine downregulates the cancer stem-like cell population.

### 3.5. Combination Treatment of AMG with Gemcitabine Increases Mitochondrial ROS Production in SNU-478 Cells

**Figure 5:**
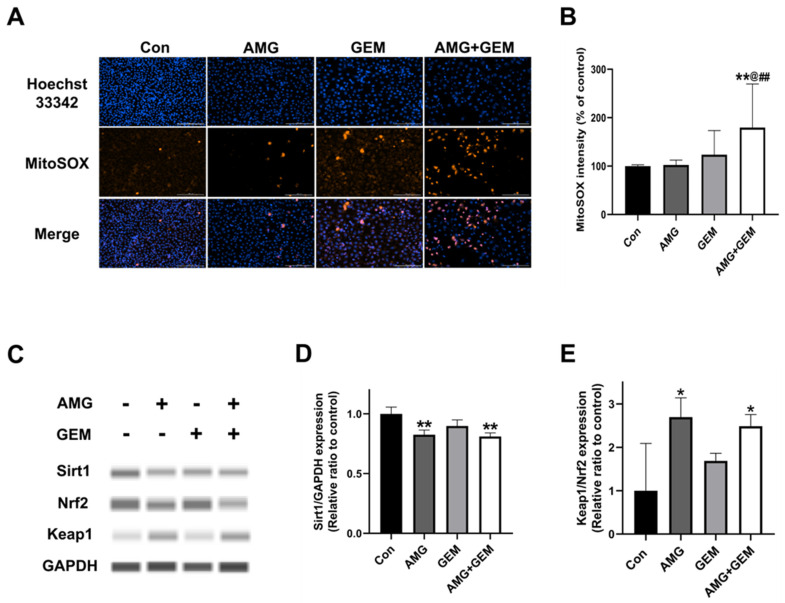
Effects of co-treatment of AMG and gemcitabine on mitochondrial ROS production. (**A**) The representative images show ROS formation using MitoSOX^TM^ and Hoechst 33342 in SNU-478 cells. Bar = 200 μm. (**B**) Quantification of MitoSOX fluorescence intensity. (**C**) The protein expression of Sirt1, Nrf2, and Keap1 was detected using Western blotting. (**D**) The Sirt1 protein expression was normalized with GAPDH. (**E**) The Keap1/Nrf2 protein expression ratio was assessed. Data are presented as means ± S.D. from at least three independent experiments. * *p* < 0.05, ** *p* < 0.01 vs. Con; ^@^ *p* < 0.05 vs. GEM; ^##^ *p* < 0.01 vs. AMG (Dunnet’s test). Con, control; AMG, amurensin G; GEM, gemcitabine.

The Sirt1/Nrf2/Keap1 signaling pathway plays a crucial role in cellular signal transduction related to oxidative stress. This pathway is activated to help cells respond to oxidative stress and maintain survival. Sirt1 inhibits the activation of Keap1, activating Nrf2, which increases anti-oxidant molecules, reducing the accumulation of ROS in the cell. To investigate the effect of AMG on ROS production in SNU-478 cells, MitoSOX^TM^ staining was performed. Combination treatment of AMG (5 μM) with gemcitabine (500 nM) significantly increased MitoSOX fluorescence intensity, compared with all other groups (Figure 5A,B). In addition, the expression of proteins related to the Sirt1/ Nrf2/ Keap1 signaling was examined, using Western blot (Figure 5C). Treatment of AMG significantly decreased the level of Sirt1, compared with the control (Figure 5D). Treatment of AMG significantly increased the Keap1/Nrf2 expression ratio, compared with the control (Figure 5E). These results indicated that co-treatment of AMG with gemcitabine increased mitochondrial ROS production in CCA, partly mediated by the downregulation of Sirt1/Nrf2 signaling by AMG.

## 4. Discussion

The CSCs are a small subset of tumor cells that have the capability of self-renewal, differentiation, and tumor recurrence. Due to these characteristics, CSCs are responsible for tumor initiation, metastasis, relapse, and chemoresistance in CCA [2,20]. Commonly, CSCs are known to comprise approximately <3% of total cells in most solid cancers, including breast cancer and colon cancer [21]. However, 20–30% of total tumor mass in CCA exhibits expression of CSC markers, which can increase the risk of tumor progression and recurrence associated with poor prognosis of CCA [1]. CSCs have unique cell surface markers in each cancer type. In CCA, CSCs exhibit specific cell markers such as CD44, CD24, CD133, and the epithelial cell adhesion molecule (EpCAM) [2]. Current data indicate that gemcitabine treatment induced the CSC population with an increased CD44^high^ CD24^high^CD133^high^ cell population. These results are partially consistent with previous studies, which show that low doses of gemcitabine increase CSCs, and increased CSCs cause higher resistance and tumor growth [22]. During the co-treatment of gemcitabine with AMG, AMG reduced CD44^high^CD24^high^CD133^high^ populations induced by gemcitabine, and significantly downregulated Sox-2 expression, in the current study. These results suggest that AMG downregulates the cancer stem-like properties mediated by gemcitabine.

In CCA, the migratory ability is one of the most distinctive characteristics associated with invasiveness, metastasis, and chemoresistance [2]. A previous study has shown that CD44 reduction is associated with proliferation, migration, and invasion in most solid tumors [23]. In addition, as a primary regulator of stemness, Sox-2 accelerates the acquisition of resistance characteristics, including tumor aggressiveness, chemoresistance, and EMT [24]. Our data showed that AMG inhibits migratory ability in CCA cells, which might be associated with the reduction in CD44 and Sox-2 expression by AMG. Moreover, CD44 is associated with ROS production, by regulating the redox status [25]. Low levels of ROS can promote tumor formation and progression by stimulating cell proliferation, angiogenesis, survival, and invasion [26]. However, high amounts of ROS can trigger cell death by inducing oxidative stress and apoptosis. In the present study, the combination treatment of AMG with gemcitabine increased mitochondrial ROS production, resulting in apoptotic death of SNU-478 CCA cells. Previous studies have reported that a high level of CD44 expression enhances the defense against ROS in gastrointestinal cancer, and the deletion of CD44+ CSC populations suppresses tumor growth [27]. Gemcitabine was reported to eliminate CSCs by increasing ROS formation [28], but the current results showed that gemcitabine treatment did not result in a significant increase compared with the control, a fact which may be related to the increase in CD133. CD133 is a pentaspan transmembrane protein associated with stem cell regeneration, differentiation, and metabolism [29]. There is evidence that gemcitabine increases the CD133 expression in pancreatic cancer, and the increasing CD133 expression inhibits the accumulation of ROS [30]. Therefore, the downregulation of the CSC subpopulation by AMG might contribute to increases in ROS formation in the present study.

**Figure 6:**
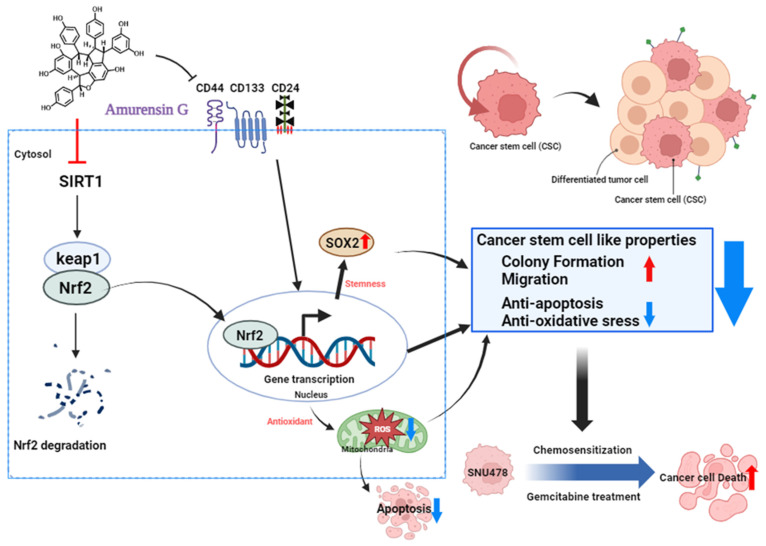
Schematic model of the mechanism of action upon treatment with AMG in SNU-478 cells (created with BioRender.com). Treatment of AMG reduces colony formation and cell migration, by inhibiting cancer stem-like properties. Inhibition of Sirt1 reduces the expression of Nrf2, leading to apoptosis through increased ROS formation. Additionally, AMG enhances cell death by sensitizing CCA to gemcitabine.

AMG is known as a potent natural Sirt1 inhibitor [31]. Sirt1 is commonly found in the nucleus of almost all cells, and regulates various cellular functions, including differentiation, proliferation, and stemness [32,33,34]. In particular, activation of Sirt1 regulates the redox system via the upregulating of the Nrf2-mediated anti-oxidant system [35]. Activation of Sirt1 activates Nrf2; this activated Nrf2 enters the nucleus without binding to Keap1, and starts transcription, which increases anti-oxidants within the cell [35]. In the current study, AMG significantly inhibits Sirt1 and Nrf2 expression and increases Keap1, which is associated with a significant increase in mitochondrial ROS by the co-treatment of AMG with gemcitabine (Figure 6). Previous studies have shown that an excessive accumulation of ROS induces a decrease in the mitochondrial membrane potential, which leads to a reduction in the Bcl-2 expression, and an increase in Bcl-2-associated X protein (BAX) and c-cas3 expression, thus resulting in apoptosis in CCA [36]. In the present study, AMG increases apoptosis in CCA cells by decreasing Bcl-2 and through an increase in c-cas3 expressions. These results suggest that the inhibition of Sirt1 by AMG might contribute to increases in apoptotic death, through an increase in mitochondrial ROS production.

Phytochemicals act through multiple pathways, making it difficult to identify the specific pathway of interest. This study was conducted at a level that suggests the possibility that AMG can reduce CCA by enhancing the anti-cancer properties of gemcitabine. Therefore, further studies will be needed to determine a clear signaling pathway, and in vivo studies are necessary to confirm the therapeutic effects of AMG and to select the optimum dose, based on clinical correlation. 

## 5. Conclusions

The current study demonstrated that AMG enhanced the chemotherapeutic ability of gemcitabine by targeting CSCs; this is associated with the upregulation of apoptosis and oxidative stress and the downregulation of migratory potential in CCA cells. Based on these results, it can be concluded that AMG may be a promising agent for CCA therapy.

## Figures and Tables

**Table 1:** List of antibodies used in Western blot assay.

Protein	Manufacturer	Molecular Weight (kDa)	Source	Clone	Dilution Ratio
Bcl-2	BioLegend	22, 26	Mouse	100	1:100
c-cas3	Cell Signaling Technology	17, 19	Rabbit	Polyclonal	1:100
Sox-2	Cell Signaling Technology	35	Rabbit	D6D9	1:100
Sirt1	Cell Signaling Technology	120	Mouse	1F3	1:100
Nrf2	Cell Signaling Technology	97–100	Rabbit	D1Z9C	1:100
Keap1	Cell Signaling Technology	60–64	Rabbit	D6B12	1:100
GAPDH	Abcam	40.2	Mouse	6C5	1:200
β-actin	Cell Signaling Technology	45	Rabbit	13E5	1:200

## Data Availability

Data are contained within the article.

## Abbreviations

AMG, Amurensin G; ANOVA, One-way analysis of variance; APC, Allophycocyanin; BAX, Bcl-2 associated X; Bcl-2, B-cell lymphoma 2; CCA, Cholangiocarcinoma; c-cas3, cleaved caspase 3; CD, Cluster of differentiation; CSC, Cancer stem cell; DPBS, Dulbecco’s phosphate-buffered saline; EMT, Epithelial-mesenchymal transition; EpCAM, Epithelial cell adhesion molecule; FACS, Fluorescence-activated cell sorting; FBS, Fetal bovine serum; FITC, Fluorescein isothiocyanate; GAPDH, Glyceraldehyde-3-phosphate dehydrogenase; HEPES, 4-(2-hydroxyethyl)-1-piperazineethanesulfonic acid; IC_50_, Half-maximal inhibitory concentration; KCLB, Korean cell line bank; Keap1, Kelch-like ECH-associated protein 1; MitoSox, Mitochondrial superoxide; Notch, Neurogenic locus notch homolog protein1; Nrf2, Nuclear factor-like 2; PE, Phycoerythrin; PI, Propidium iodide; ROS, Reactive oxygen species; RPMI, Roswell Park Memorial Institute; S.D., Standard deviation; Sirt1, Sirtuin1; SNU, Seoul National University; Sox-2, SRY-BOX transcription factor; Wnt, Wingless-related integration site; WST, Water-soluble tetrazolium salt.
